# The books are alive with biological data: an introduction to the field of biocodicology and its implications for historical health sciences collections

**DOI:** 10.5195/jmla.2021.1080

**Published:** 2021-04-01

**Authors:** Megan Rosenbloom

**Affiliations:** 1 meganrosenbloom@library.ucla.edu, Collection Strategies Librarian, UCLA Library, University of California Los Angeles, Los Angeles, CA

**Keywords:** biocodicology, history, rare books

## Abstract

Recent global events have underscored the need for broad access to digitized library special collections. At the same time, a burgeoning field of scientific and historical inquiry is finding a goldmine of data in the physical old books and manuscripts stored for centuries on library shelves. This article gives an overview of some of the interesting studies employing library materials in the new field of biocodicology, which expands the field of codicology (learning about book history through studying a copy's physical attributes, sometimes referred to as “archaeology of the book”) to interrogate physical books with proteomic, genomic, and microbiomic tools. Historical health sciences collections provide rich, new research avenues for budding biocodicologists, and biocodicology and other interdisciplinary fields focused on material culture present an unforeseen justification for institutions' continued preservation and access to individual physical copies.

Over the past few decades, libraries have reaped manifold benefits from digitizing library special collections. Digital surrogates help preserve original items by diminishing handling, give researchers more robust searching and comparison capabilities, and enable the creation of visually rich online exhibitions and social media. During the global coronavirus pandemic, access to these digital collections has become even more valued to bolster remote teaching, learning, and research. However, there are still many scholarly pursuits with special collections that cannot be replicated online, and the new field of biocodicology offers promising avenues of inquiry using physical copies of old books in previously inconceivable ways to uncover truths about the past. Though few historical health sciences collections have been subjected to biocodicological inspection, books in medical libraries and museums hold special promise for this burgeoning field.

Codicology—from the Latin “codex,” meaning book, and usually referring to handwritten books—studies the physical structure of books to learn about their history and manufacture. With biocodicology, biomolecular tools unveil books' proteomic (proteins), genomic (DNA), and microbiomic (microorganisms and their genes) data. Revolutionary advances in these fields in the past decade have allowed the mass collection and analysis of these data, an approach that pioneering biocodicologist Sarah Fiddyment said, “has the advantage of detecting possibly surprising elements that would not be identified in a more targeted approach, leading to unexpected discoveries. It also gives a more representative assessment of the environment analysed and can allow for relative quantification of identified elements” [1]. The field of biocodicology is so new that the first conferences and workshops appear to have taken place in 2018 and 2019 [2].

A number of the formative studies in biocodicology focus on analyzing parchment from medieval books. A team led by the aforementioned Fiddyment has developed a minimally invasive sample collection method where erasers are rubbed on the surface of parchment, and then the protein trapped in the eraser crumbs is digested with an enzyme. The peptides thus produced are used to determine the animal of origin of the ultrafine parchment known as uterine vellum. The team was able to use their results to suggest that the thinness of the parchment was an innovation achieved by a technological process on cow skin and not by employing other rumored animals such as squirrels or rabbits [3].

This kind of work can reveal historical insights into medieval livestock economies and craft techniques about which no contemporary written records exist. Analysis of the *York Gospels*, believed to have been written in Canterbury, England, in the tenth century CE, revealed “a rich palimpsest of biological information from a complete book object of great cultural value over its 1000-year history,” including evidence of a rinderpest (or cattle plague) outbreak that resulted in a bumper crop of hides for parchment. This team believed further analysis of the data could even reveal the colors of the animals' coats and their ages [4].

Biocodicology is not only useful for learning about the oldest books and manuscripts. One parchment fragment alleged to be part of New Zealand's founding document, the Treaty of Waitangi of 1840, was shown to be legitimate due to data showing both pieces of parchment being made from ewes with matching mitochondrial genomes, even though the size and characteristics of the two pieces did not appear to match visually. Researchers were then able to trace the differences in appearance to conservation work that took place in the 1970s and 1980s [5]. Here, biocodicology contributed to the verification of historical documents and the ability to retrace the steps of past conservators of world heritage objects, providing valuable lessons for future conservation.

The Anthropodermic Book Project's work could be considered an early form of biocodicology as well. Instead of focusing on parchment, to date, the team has focused on alleged human skin leather–bound books and analyzed thirty-one of the fifty that we could identify in public institutions' collections. We analyzed samples from the books' bindings using a proteomic method called peptide mass fingerprinting (PMF), which allowed us to identify the protein collagen and match the binding material to its mammalian source. Eighteen of the book bindings were confirmed as human skin [6]. Once confirmed scientifically, provenance research on these books revealed that most of the books with real human skin had doctor bibliophiles complicit in their creation, exposing uncomfortable facts about the history of clinical medicine and the way doctors viewed and used their patients' bodies.^*^ The PMF test employed by the Anthropodermic Book Project is straightforward, sensitive, and specific enough to put to rest some long-held rumors in the library field and confirm others.

More scientifically complex microbiomic tests have even more to reveal to those interested in the history of medicine. Three papers by analytical chemist Alfonsina D'Amato and her collaborators plumb such depths of data that their claims almost sound like the stuff of science fiction. Milanese death registries of 1630 captured evidence of the plague ripping through the city thanks to seventeen strains of Yersiniaceae family proteins, evidence of dozens of contacts with humans and mice, and a surprisingly large amount of plant traces like potatoes, corn, rice, carrot, and chickpeas that purportedly represent what the scribes ate for lunch while they filled out the death tolls. D'Amato's data really paint a picture of the world in which this book was created: “It would appear that, whereas the scribes sweated all day long to register the very large number of dying persons, during the night the same [sample sites] were explored by hordes of rats looking for food” [8].

Postcards, letters, and the death-bed attire of Russian playwright Anton Chekhov denoted the presence of the tuberculosis bacteria believed to have killed him [9]. Another study examined the manuscript of Mikhail Bulgakov's *Master i Margarita*, pointing to biomarkers of nephrotic syndrome and the presence of morphine, which one historian theorized was planted by Stalin's KGB [10]. Who knew the microbiomic worlds of old books and manuscripts could be filled with such intrigue? What privacy and ethical quandaries might these kinds of studies one day unleash?

A monk's kiss on a gospel, a bookworm's excrement [11], lapis lazuli in the dental calculus of a woman's skeleton alluding to her work as a medieval manuscript artist [12]: exploring these minute pieces of evidence underlines what one interdisciplinary project calls “the contaminated book as a cultural good” and transforms “dead” collections into “living” ones [13]. The world's health sciences library and museum collections have a wealth of potential research material for the biocodicologist. An informal survey of the Librarians, Archivists, and Museum Professionals in the History of the Health Sciences (LAMPHHS)^†^ put forth a variety of possibly interestingly contaminated objects in their collections, including a 1920 lab book (bubonic plague), Sir Alexander Fleming's microscope (penicillin), Louis Pasteur's letters (rabies) [14], a leprosy doctor's notebook (thalidomide) [15], and nineteenth-century dissecting room books (one can only guess) [16].

This avenue of research using physical special collections was unimaginable until very recently and underscores the importance of the preservation of and access to the physical book in addition to its digital surrogate. Interdisciplinary fields such as biocodicology, material culture, heritage science, and the history of the book place a new emphasis on libraries' responsibilities to preserve their individual copies and all of the unique markings and data they may contain. Book historian David Pearson imagined in the near future that “a twenty-first-century undergraduate textbook which has been covered with highlighter pen and marginal scribble may one day be valued more highly than the clean copy that some today might prefer” [17]. As our culture moves further away from the printed book, interest in the evidence provided in physical copies will likely continue to grow, and the tools with which they are studied will certainly continue to mature. Stewards of historical health sciences collections would do well to attempt to anticipate these uses to help keep their collections alive for future researchers.

## Footnotes

* For a book-length exploration of this theme, see Rosenbloom [7].

† As of 2020, LAMPHHS is the combined entity of two former groups: Archivists and Librarians in the History of the Health Sciences (ALHHS) and Medical Museums Association (MeMA).
